# A post-translational modification signature defines changes in soluble tau correlating with oligomerization in early stage Alzheimer’s disease brain

**DOI:** 10.1186/s40478-019-0823-2

**Published:** 2019-12-03

**Authors:** Ebru Ercan-Herbst, Jens Ehrig, David C. Schöndorf, Annika Behrendt, Bernd Klaus, Borja Gomez Ramos, Nuria Prat Oriol, Christian Weber, Dagmar E. Ehrnhoefer

**Affiliations:** 1 BioMed X Innovation Center, Im Neuenheimer Feld 515, 69120 Heidelberg, Germany; 2 B CUBE – Center for Molecular Bioengineering, Technische Universitaet Dresden, 01307 Dresden, Germany; 3 Centre for Statistical Data Analysis, European Molecular Biology Laboratory (EMBL), 69117 Heidelberg, Germany; 4 Present address: Life Sciences Research Unit, University of Luxembourg, L-4367 Belvaux, Luxembourg; 5 Present address: Luxembourg Centre for Systems Biomedicine, University of Luxembourg, L-4362 Esch-sur-Alzette, Luxembourg

**Keywords:** Alzheimer’s disease, Tau, Posttranslational modifications, Tau oligomerization

## Abstract

Tau is a microtubule-binding protein that can receive various post-translational modifications (PTMs) including phosphorylation, methylation, acetylation, glycosylation, nitration, sumoylation and truncation. Hyperphosphorylation of tau is linked to its aggregation and the formation of neurofibrillary tangles (NFTs), which are a hallmark of Alzheimer’s disease (AD). While more than 70 phosphorylation sites have been detected previously on NFT tau, studies of oligomeric and detergent-soluble tau in human brains during the early stages of AD are lacking. Here we apply a comprehensive electrochemiluminescence ELISA assay to analyze twenty-five different PTM sites as well as tau oligomerization in control and sporadic AD brain. The samples were classified as Braak stages 0–I, II or III–IV, corresponding to the progression of microscopically detectable tau pathology throughout different brain regions. We found that soluble tau multimers are strongly increased at Braak stages III–IV in all brain regions under investigation, including the temporal cortex, which does not contain NFTs or misfolded oligomers at this stage of pathology. We additionally identified five phosphorylation sites that are specifically and consistently increased across the entorhinal cortex, hippocampus and temporal cortex in the same donors. Three of these sites correlate with tau multimerization in all three brain regions, but do not overlap with the epitopes of phospho-sensitive antibodies commonly used for the immunohistochemical detection of NFTs. Our results thus suggest that soluble multimers are characterized by a small set of specific phosphorylation events that differ from those dominating in mature NFTs. These findings shed light on early PTM changes of tau during AD pathogenesis in human brains.

## Introduction

Alzheimer’s disease (AD) is the most common form of neurodegenerative diseases and is characterized pathologically by the presence of both neurofibrillary tangles (NFTs) and senile plaques [1–3]. While senile plaques are extracellular deposits of amyloid β-peptides [4], NFTs are formed intracellularly and consist of abnormally phosphorylated tau, a microtubule binding protein [5]. Mutations in the genes which affect the levels of amyloid β-peptide, such as APP (amyloid precursor protein), PSEN1 (Presenilin 1) and PSEN2 (Presenilin 2) cause familial AD (fAD) [6, 7]. On the other hand, sporadic AD (sAD), which accounts for more than 90% of all AD cases, is a multifactorial disease likely due to both genetic and environmental risk factors [8–10]. While sAD usually has a later onset compared to fAD, the disease progresses otherwise in a similar fashion [11, 12].

Both biomarker and neuropathological data show that tau pathology parallels cognitive dysfunction in AD more closely than amyloid β pathology [13, 14]. In particular, tau NFTs spread in a stereotypical manner throughout the brain, which has been used by Braak and colleagues as a method to differentiate disease stages [15]. In Braak stages I and II, which are very common in the elderly [13], NFTs are localized to the transentorhinal cortex. In Braak stages III and IV, the limbic regions such as hippocampus are additionally positive for NFTs. Finally, in Braak stages V and VI, neocortical involvement of NFTs is observed [15, 16].

While NFT formation is difficult to recapitulate in disease models and its exact cellular mechanisms remain to be further elucidated, it is well established that posttranslational modifications (PTMs) on tau protein have a role in this process [17, 18]. Tau is heavily modified in both health and disease by several different PTMs such as phosphorylation, nitration, glycosylation, methylation, acetylation, sumolyation, ubiquitination and truncation [19, 20]. Among all these different types of modifications, phosphorylation is studied most extensively [21]. Hyperphosphorylated tau molecules dissociate from microtubules and form detergent-soluble oligomeric structures, which later progress into detergent-insoluble aggregates [22]. The tau oligomer, an intermediate structure formed before the formation of NFTs, is thereby likely responsible for neuronal toxicity [23–28]. Even tau monomers were recently shown to be capable of adopting a conformation that promotes the seeding and spreading of pathology [29–31]. To analyze different tau structures, conformation-specific antibodies have been developed, which are thought to react with the different folding states of the protein: Antibodies raised against oligomeric forms of tau such as T22, TOC1 and TOMA selectively label tau oligomers over monomers [24, 25, 32], whereas Alz50 and MC1 detect PHFs and NFTs [33, 34].

To date, many studies focusing on tau PTMs were carried out either under cell-free conditions, in cultured cell lines or in animal models. These studies provided valuable information on the enzymes modifying tau, such as kinases and phosphatases, and on the consequences of these modifications. For example, phosphorylation events at the sites T231, S235, S262, S293, S324, S356 decrease the affinity of tau to microtubules and result in destabilization of the neuronal cytoskeleton [35–37], while phosphorylation at C-terminal sites such as S422 promotes tau self-aggregation and can inhibit tau truncation at D421 [38, 39]. Studies using human brains are more limited, but several tau PTMs have been identified in postmortem samples using mass spectrometry and immunohistochemistry approaches, which we summarized previously (www.tauptm.org) [19]. However, most of these studies focused on PTMs present on NFTs, since detergent-soluble, oligomeric tau is more difficult either to discern by immunohistochemistry or to purify for mass spectrometry approaches.

ELISA-based techniques, on the other hand, are quantitative and allow for the detection of tau PTMs in whole tissue lysates [40]. We have previously established a panel of validated tau antibodies covering twenty-five PTM sites [19], which we applied here to study tau PTMs in aged brains. We studied controls and sporadic AD samples ranging from Braak stages 0 to IV, and brain regions that are sequentially affected by tau pathology in AD: entorhinal cortex, hippocampus and temporal cortex. We furthermore developed an ELISA method to quantify non-monomeric tau species in detergent-soluble extracts and demonstrated that these species increase in all analyzed brain regions at Braak stages III–IV, in parallel with specific alterations in tau PTMs. Importantly, these PTMs were not changed at Braak stage II or in iPSC-derived neurons, where detergent-soluble tau multimers were also not detected. The pattern of altered tau PTMs was strikingly similar in all brain regions analyzed, which led us to define a tau PTM signature characteristic for early, disease-associated changes in AD. These results thus advance our knowledge on tau pathology and have implications for future diagnostic and therapeutic approaches targeting tau.

## Methods

### Human brain tissue lysate preparation

Anonymized human post-mortem tissues (Table 1) were obtained from the London Neurodegenerative Diseases Brain Bank, a member of the Brains for Dementia Research Network. Lysates from human entorhinal cortices, hippocampi and temporal cortices were prepared in lysis buffer containing 150 mM NaCl, 20 mM Tris pH 7.5, 1 mM EDTA, 1 mM EGTA, 1% Triton-X100 and protease, phosphatase, demethylase (500 μM IOX1 (Active Motif), 2 μM Daminozide (Active Motif), 10 μM Paragyline Hydrochloride (Sigma)), deacetylase (10 μM Trichostatin A (Sigma), 5 mM Nicotinamide (Sigma)), O-GlcNAcase (1 μM Thiamet-G (Sigma)) inhibitors. Lysis was performed with a dounce homogenizer. The homogenized lysates were spun down at 18000×g at 4 °C for 30 min. The supernatant was collected, and the protein concentration was measured by BCA assay according to manufacturer’s instructions (BioRad).

**Table 1:** List of anonymized brain samples received from Brains for Dementia Research Network. EC: Entorhinal Cortex, Hip: Hippocampus, TC: Temporal Cortex

ID	Sex	Age	Braak tangle stage	Thal phase	APOE genotype	Postmortem delay (h)	Tissues obtained	Brain bank
Ctrl1	M	78	0	0	3/4	56	EC, Hip, TC	South West Dementia Brain Bank
Ctrl2	F	86	1	1	3/3	38.5	EC, Hip, TC	South West Dementia Brain Bank
Ctrl3	F	96	1	0	3/3	49	EC, Hip, TC	South West Dementia Brain Bank
Ctrl4	M	92	1	2	3/4	56.5	EC, Hip, TC	South West Dementia Brain Bank
Ctrl5	F	70	1	0	2/4	55.5	EC, Hip, TC	South West Dementia Brain Bank
Ctrl6	F	69	0	1	3/4	48	EC, Hip, TC	London Neurodegenerative Diseases Brain Bank
Ctrl7	M	74	1	1	3/3	24	EC, Hip, TC	London Neurodegenerative Diseases Brain Bank
Ctrl8	M	77	0	0	3/3	11	EC, Hip, TC	London Neurodegenerative Diseases Brain Bank
AD1	F	83	2	N/A	3/4	39	EC, Hip, TC	London Neurodegenerative Diseases Brain Bank
AD2	M	80	2	1	3/3	31	EC, Hip, TC	London Neurodegenerative Diseases Brain Bank
AD3	F	76	2	N/A	3/3	22	EC, Hip, TC	London Neurodegenerative Diseases Brain Bank
AD4	F	86	2	1	3/3	45	EC, Hip, TC	London Neurodegenerative Diseases Brain Bank
AD5	M	96	3	3	3/3	25.5	EC, Hip, TC	South West Dementia Brain Bank
AD6	F	85	3	0	2/2	13.5	EC, Hip, TC	South West Dementia Brain Bank
AD7	F	95	3	4	2/4	65.5	EC, Hip, TC	South West Dementia Brain Bank
AD8	F	87	4	4	3/4	71.5	EC, Hip, TC	South West Dementia Brain Bank
AD9	M	81	4	5	3/4	38	EC, Hip, TC	South West Dementia Brain Bank

### Electrochemiluminescence ELISA

Meso Scale Discovery (MSD) Gold Streptavidin small-spot 96-well plates were blocked with 5% (w/v) Blocker A solution in Tris wash buffer (50 mM Tris-HCl pH 7.5, 150 mM NaCl and 0.02% Tween-20). Plates were sealed and allowed to block for 1 h at room temperature (RT) on a plate shaker. The plates were then washed three times with Tris wash buffer and coated with 25 μL of biotinylated antibody diluted in 1% Blocker A solution. The biotinylation of the antibodies was performed according to the manufacturer’s instructions (EZ-Link Sulfo-NHS-Biotin, Cat No. 21217, Thermo Scientific). Before biotinylation, BSA was removed with the Melon Gel IgG Purification Kit (Cat. No 45212, Thermo Scientific), if necessary. After incubating for 1 h at RT on a plate shaker, plates were washed three times with Tris wash buffer. For each sample 1 μg of protein lysate (diluted in 50 μl 1xTBS) was incubated for 1 h at RT on a plate shaker. For analysis of denatured samples, samples were boiled in SDS-containing buffer (62.5 mM Tris-HCl pH 6.8, 10% Glycerol, 2% SDS) where the final amount of detergent did not exceed 0.02%. Plates were washed three times with Tris wash buffer to get rid of unbound lysates and then incubated with 25 μl of 0.5 μg/ml detection antibody (Tau12 labeled with MSD Sulfo-Tag-NHS-Ester, Cat. No: R31AA, Meso Scale Discovery) diluted in 1% Blocker A solution for 1 h at RT on a plate shaker. The plates were then washed three times with Tris wash buffer. 150 μl of 2X Read Buffer (Cat. No. R92TC, Meso Scale Discovery) were added 5 min before the signal was measured on a Meso Scale Discovery Quickplex platform.

### Antibodies

The antibodies used in this study were characterized previously [19]. Information on the supplier and catalog numbers can be found in Table 2.

**Table 2:** List of tau antibodies used in this study

Name	Species	Company	Cat No.
Tau12	mouse	Biolegend	SIG-39416
Tau5	mouse	Abcam	ab80579
Tau1	mouse	Millipore	MAB3420
Dako	rabbit	Agilent Dako	A0024
BT2	mouse	Thermo Fisher	MN1010
HT7	mouse	Thermo Fisher	MN1000
T22	rabbit	Sigma-Aldrich	ABN454-I
nY18	mouse	Biolegend	829,701
nY29	mouse	Millipore	MAB2244
acK280	rabbit	Anaspec	AS-56077
meK311	mouse	Biolegend	MMS-5102
C3-D421	mouse	Millipore	36–017
pY18	mouse	Novusbio	NBP2–42402
pT181	rabbit	Thermo Fisher	701,530
pS198	rabbit	Abcam	ab79540
pS199	rabbit	Thermo Fisher	701,054
pS202	rabbit	Anaspec	AS-28017
pS199/202	rabbit	Thermo Fisher	44-768G
pT205	rabbit	Abcam	ab181206
pT212	rabbit	Abcam	ab51053
pS214	rabbit	Thermo Fisher	PA5–35762
pT217	rabbit	Thermo Fisher	44–744
pT231	rabbit	Thermo Fisher	701,056
pS235	rabbit	Thermo Fisher	PIPA535761
pS238	mouse	Abcam	ab128889
pS356	rabbit	Abcam	ab51036
pS396	rabbit	Thermo Fisher	44-752G
pS400	rabbit	Anaspec	AS-54978
pS404	rabbit	Thermo Fisher	44-758G
pS409	rabbit	Abcam	ab4861
pS416	rabbit	Abcam	ab119391
pS422	rabbit	Abcam	ab79415

### Statistical analysis of ELISA data

Total tau intensity values were scaled within each sample type by dividing them by their geometric mean. The data was then normalized by the dividing the background-corrected signal intensity by the scaled total tau values. Subsequently, we used the generalized logarithm on the log2 scale to put our normalized values on the log2-scale [41]. We then removed all normalized values below 0, which correspond to signal intensities below the background range.

We performed a differential analysis using the software package *limma* [42, 43]. For this, we created a design matrix that compares the fold change between the AD and control conditions within each of the tissues. In total, we performed 4 comparisons: EC-Braak-II vs. EC-Braak-0–I, EC-Braak-III–IV vs. EC-Braak-0–I, Hip-Braak-III–IV vs. Hip-Braak-0–I, TC-Braak-III–IV vs. TC-Braak-0–I. Statistical significance was determined with an “omnibus” test (similar to an ANOVA procedure) to determine overall differences within the dataset and applied a FDR cutoff of 5% to obtain a list of candidate PTMs. Finally, individual comparisons within each tissue type were performed to determine the location of the change.

### Recombinant tau protein purification

Tau variants (full length protein and a fragment encoding amino acids 256–368) were cloned into the pET19b vector (Novagen) in between the NcoI and BamHI restriction sites. The pET19b-Tau plasmids were transformed into *E. coli* BL21(DE3) cells (Novagen). Cells were grown in LB supplemented with ampicillin at 37 °C until OD600 reached 0.6–0.8. The expression of the tau proteins was induced by the addition of 1 mM IPTG. The cells were then grown for an additional 3 h at 37 °C and harvested by centrifugation. The cell pellet was resuspended in running buffer (50 mM Na-phosphate pH 7.0, 1 mM EGTA and 1 mM DTT) supplemented with cOmplete protease inhibitors (Roche), benzonase (Merck) and 10 μg/ml lysozyme (Sigma). The cells were lysed by 4 passages through an EmulsiFlex C3 (Avestin). After centrifugation and filtration, the cleared lysates were boiled for 20 min at 100 °C. After another centrifugation and filtration step the lysate was then loaded onto a combination of a HiTrap Q and a HiTrap SP column (GE Healthcare) pre-equilibrated with running buffer. After loading the sample, the HiTrap Q column was removed. The HiTrap SP column was washed with running buffer and eluted in a gradient to running buffer containing 300 mM NaCl. The HiTrap SP elution fractions containing the tau proteins were concentrated using a 30 MWCO or 3 MWCO Amicon centrifugal filter unit (Merck) and loaded on a HiLoad 16/600 Superdex 75 pg size exclusion chromatography column (GE Healthcare) equilibrated with running buffer. After SDS-PAGE analysis, the elution fractions with the highest purity were pooled and quantified. The samples were aliquoted, flash-frozen in liquid nitrogen and stored at − 80 °C.

### Tau aggregation assay

Aggregation of tau proteins was evaluated with a thioflavin T assay. 10 μM of tau protein was mixed with 20 mM Tris pH 7.5 containing 100 mM NaCl, 1 mM EDTA, 1 mM DTT, 0.03 mg/mL heparin sodium salt and 30 μM thioflavin T. Aggregation signal was measured every 30 min for a total duration of 40 h using a fluorescence plate reader (EX: 450 nm, EM: 520 nm) at 37 °C. In parallel, vials containing the same aggregation mix without thioflavin T were incubated at 37 °C for indicated time points. Samples were then flash-frozen in liquid nitrogen before storage at − 80 °C. These samples were used for electrochemiluminescence analysis as follows: aggregation samples were thawed, sonicated for 30 s and diluted in 1X TBS. The samples were either boiled or not boiled in SDS-containing buffer (62.5 mM Tris-HCl pH 6.8, 10% Glycerol, 2% SDS) for 10 min as indicated, the final amount of detergent in the sample did not exceed 0.02%. 100 pg of tau aggregation sample were added per well of an MSD Gold Streptavidin small-spot 96 well plate (Meso Scale Discovery). ELISA analysis was then performed as described above and previously [19].

### Immunoprecipitation of tau from EC lysates

100 μg of entorhinal cortex lysates from Braak 0–I and Braak III–IV were used for immunoprecipitation with Tau12 antibody. Magnetic Protein G beads (Dynabeads, Thermo Fisher) were blocked with Pierce protein free TBS blocking buffer and the beads were incubated with 8 μg of Tau12 antibody for 1 h at RT. The beads were washed with lysis buffer and incubated with 100 μg of EC lysates overnight at RT. Next day, beads were washed with lysis buffer and bound protein was eluted with 100 μl of 50 mM Glycin pH 2.8 and the pH was neutralized with Tris.

### Atomic force microscopy

Cluster sizes of tau oligomers were measured with atomic force microscopy (AFM). Braak 0–I and Braak III–IV entorhinal cortex Tau12-IP eluates were deposited on freshly cleaved mica sheets and incubated for 60 min in a closed chamber with 100% humidity to avoid evaporation. The samples were then washed by 5x buffer exchange with Tris buffer (50 mM Tris pH 7.5, 150 mM NaCl). Atomic force microscopy measurements were carried out with a NanoWizard4 AFM (JPK, Germany) operated in the “QI Advanced Imaging” mode using BL-AC40TS cantilevers (Olympus, Japan). Cantilevers were calibrated using the automatic “contact-free” method of the JPK NanoWizard Control software. AFM images were acquired of 1 × 1 μm^2^ areas using a setpoint of 0.2 nN, a z-length of 100 nm and a pixel time of 6 ms. The “measured height” data were saved and further processed in the Gwyddion software (ver. 2.53) [44] as follows. Line levelling was done by subtracting first-order polynomial fits from each scan line – for this, larger features of the image were masked. To remove noise, the “conservative denoise” and the “Gaussian” filter were applied to the images with their “size”-parameters set to 3 and 2 pixels, respectively. Cluster detection was carried out using the “Interactive H_Watershed” plugin from the “SCF MPI CBG” repository [45] of the software Fiji [46]. For each detected cluster the maximum height value was saved and statistics on all cluster heights were then obtained using the software MATLAB (MathWorks).

### Generation of hiPSC-derived neurons

Donor information as well as cell line identifiers are summarized in Additional file 1: Table S1. iPSC lines Ctrl3, sAD3, fAD1, fAD2, fAD3 and fAD4 were obtained from StemBancc. Ctrl1, Ctrl2, sAD1 and sAD2 were generated using ReproRNA technology (Stem Cell Technologies) and characterized in detail elsewhere [47]. All iPSCs were differentiated into neurons following a cortical neuronal induction protocol [48] with minor modifications. iPSC colonies were dissociated using Versene (Invitrogen) and seeded at a density of 200,000 cells/cm^2^ in mTesR (Stemcell Technologies) with 10 μM Rock inhibitor (SelleckChem). The next day, the medium was switched to neural induction medium containing N2B27 Medium (50% DMEM/F 12, 50% Neurobasal, 1:200 N2, 1:100 B27, 1% PenStrep, 0.5 mM Non-essential amino acids, (all Invitrogen), 50 μM ß-mercaptoethanol (Gibco), 2.5 μg/ ml insulin and 1 mM sodium pyruvate (both Sigma)), 10 μM SB431542 (Selleckchem) and 1 μM Dorsomorphin (Tocris) and changed daily for 11 more days. On day 12, cells were split using Accutase (Invitrogen) to a density of 220,000 cells/cm^2^ in N2B27 Medium containing 10 μM Rock inhibitor and 20 ng/ml FGF2 (Peprotech). The medium was changed every third day without Rock inhibitor. On day 25, cells were split using Accutase to a density of 220,000/cm^2^ in final maturation medium containing N2B27 medium with 20 ng/ml BDNF, 10 ng/ml GDNF (both Peprotech), 1 mM dibutyryl-cAMP (Sigma), 200 μM ascorbic acid (Sigma) and 10 μM Rock inhibitor (SelleckChem). The medium was changed every third day without Rock inhibitor until day 60.

### Microscopy

iPSC derived neurons were seeded at day 40 in a density of 20,000 cells/well on a 96-well imaging microplate (Greiner) and fluorescence pictures were taken between day 50–60. For imaging, cells were washed once with PBS and fixed with 4% PFA (Fisher Scientific) for 20 min at room temperature. Cells were permeabilized with 0.1% Triton X-100 (Sigma) in PBS for 10 min and blocked with 5% BSA (Sigma) in PBS for 1 h RT at room temperature. Primary antibodies were diluted in 5% BSA in PBS and cells were incubated over night at 4 °C. The next day, cells were washed 3x with PBS and incubated with secondary antibodies for 1 h at room temperature in the dark. Afterwards, cells were washed again 3x with PBS and imaged with an Axio Observer D1 (Zeiss). Antibodies used for microscopy analysis of iPSC-derived neurons were: MAP 2 (Biolegend, 822,501), GABA (Sigma, A2052), NeuN (Sigma, MAB377), VGlut1 (Thermo Scientific, 48–2400), Tuj1 (Cell Signaling Technologies, 4466), Tbr1 (Abcam, ab183032).

## Results

In this study, we used Triton-X100-soluble fractions from entorhinal cortices (EC), hippocampi (Hip) and temporal cortices (TC) from the same patients (Braak stages 0–I and III–IV) to monitor differences in Tau PTMs between brain regions sequentially affected by tauopathy in AD. We additionally analyzed the EC, Hip and TC from donors classified as Braak II to investigate whether alterations in Tau PTMs would already be apparent at this stage. Donors from all groups were within the same age range (69–96 years, Table 1).

To detect changes in tau PTMs quantitatively, we used a previously established electrochemiluminescence ELISA assay, with a validated tau PTM antibody panel [19] (Table 2). Briefly, this consists of a sandwich ELISA approach, with PTM-specific tau capture antibodies and Tau12, a total tau antibody, for detection. We quantified a total of twenty-five PTM sites: nitrated tyrosine 18 (nY18) and nitrated tyrosine 29 (nY29), acetylated lysine 280 (acK280), methylated lysine 311 (meK311), caspase cleaved tau at aspartic acid 421 (C3-D421) and twenty phosphorylation sites, including one tyrosine (pY18), five threonine (pT181, pT205, pT212, pT217, pT231) and fourteen serine (pS198, pS199, pS199 + 202, pS202, pS214, pS235, pS238, pS356, pS396, pS400, pS404, pS409, pS416, pS422) modifications (Table 2). We then normalized the PTM signals to total tau determined with the Tau5-Tau12 ELISA pair. However, it is important to note that comparisons across different sites (antibodies) should be avoided due to potential differences in biotinylation efficiencies and binding affinities of the antibodies.

### Native Braak III–IV, but not Braak II brain extracts show extensive changes in all tau PTMs analyzed

**Fig. 1:**
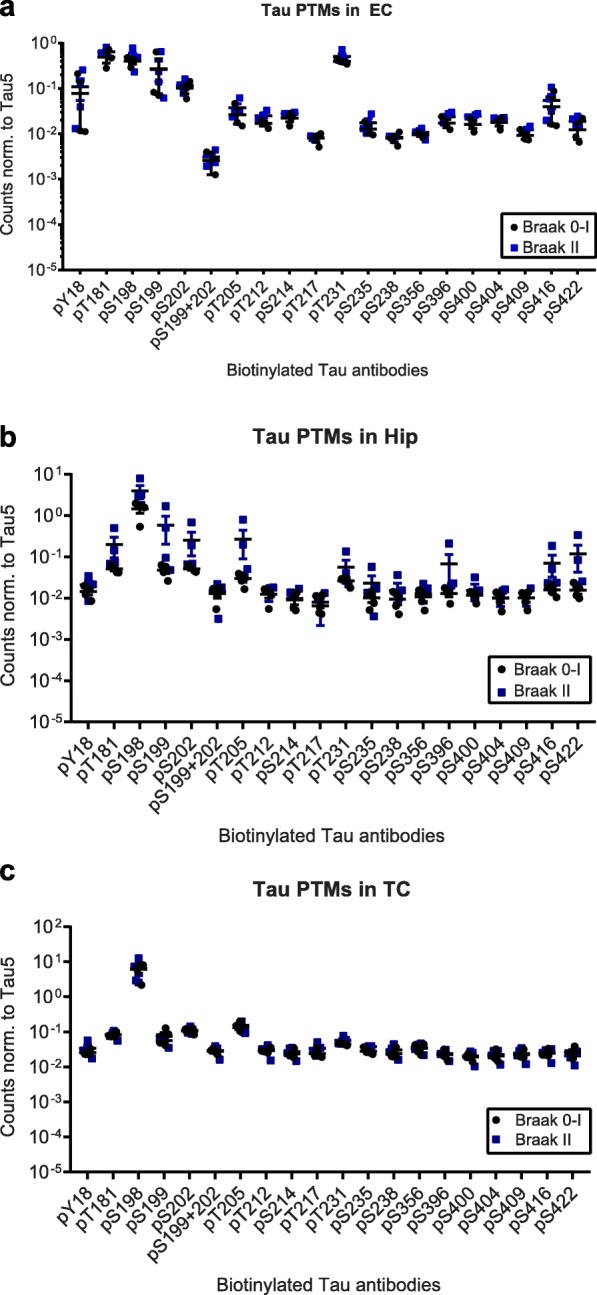
Tau phosphorylation does not change in Braak II samples compared to Braak 0–I controls. Normalized phospho-tau signals from Braak II and Braak 0–I **a)** entorhinal cortices (EC), **b)** Hippocampi (Hip) and **c)** Temporal cortices (TC). Biotinylated antibodies were used as capture, sulfo-tagged Tau12 was used for detection. None of the observed changes were significant (*p* > 0.05, t-tests)

First we compared tau PTMs in the EC, Hip and TC from donors classified as Braak 0–I to those classified as Braak II [13]. While PTMs were present in all samples under investigation (Fig. 1 and Additional file 2: Figure S1), fold changes were small and not significant.

**Fig. 2:**
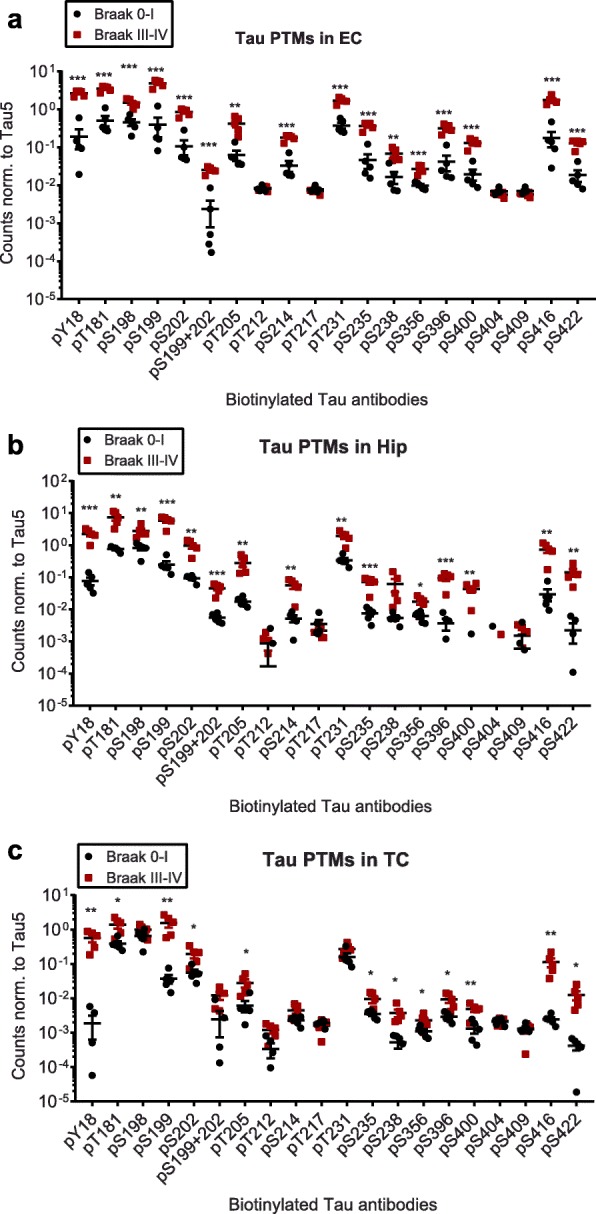
Compared to Braak 0–I samples, many but not all, tau phosphorylation events are increased in native Braak III–IV samples. Normalized phospho-tau signals obtained from ELISA measurements of samples from **a**) entorhinal cortices (EC), **b**) hippocampi (Hip) and **c**) temporal cortices (TC). Student’s t-tests: *, *p* < 0.05, **, *p* < 0.01, ***, *p* < 0.001

We therefore moved on to the comparison between Braak stages 0–I and III–IV, where we investigated tau PTMs in the EC, Hip and TC from the same donors. In this analysis, both EC and Hip tissues derived from Braak stages III–IV showed an increase in phosphorylation at most sites, with the exception of pT212, pT217, pS404 and pS409 (Fig. 2 a and b). In TC, this set of four was among the eight sites unaltered in Braak III–IV patients, while 12 sites were also significantly increased in this tissue (Fig. 2c). Among the non-phospho PTMs that are part of our panel [19], only cleavage at D421 was increased in all three brain regions, while nitration at Y18 showed a significant increase in the EC (Additional file 3: Figure S2). Although this reflects the expected severity of tauopathy in the different brain regions (EC > Hip > TC), we were concerned that potential soluble tau oligomers may influence ELISA signals when an assembly containing more than one tau molecule is bound by each capture antibody. We therefore analyzed whether any multimeric tau structures were present in our samples.

### Triton-X100-soluble brain fractions contain tau multimers and Braak III–IV ECs contain more of multimeric tau structures with heights differing between 10 nm - 30 nm

For the analysis of tau multimers in detergent-soluble brain extracts we established an ELISA that uses Tau12 both as the capture and the detection antibody. In monomeric tau, the Tau12 epitope will be blocked upon binding to the capture antibody and, as a consequence, the detection antibody will not be able to bind and no signal will be generated. In contrast, multimeric tau contains additional, free Tau12 epitopes on other tau molecules in the same structure and thus will give a signal. Such an approach of using monoclonal antibodies raised against tau has been previously applied to detect multimeric species [40, 49]. During the oligomerization and aggregation process, tau furthermore undergoes a conformational shift which has been associated with toxicity and can be detected with conformation-specific antibodies such as T22 ([25, 50]).We therefore set up an additional ELISA method to detect oligomers containing misfolded tau using the conformation-specific antibody T22 as a capture and Tau12 for detection.

**Fig. 3:**
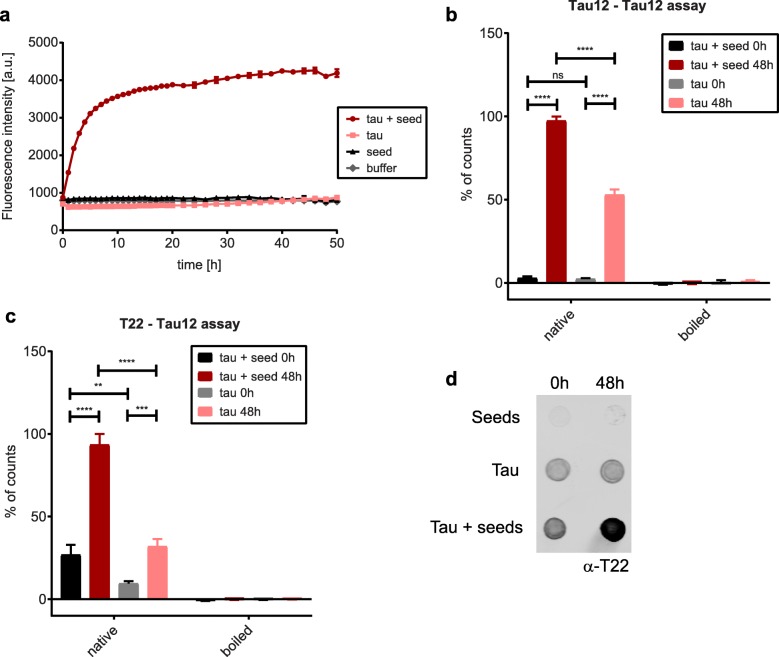
Oligomerization of tau can be monitored with Tau12-Tau12 or T22-Tau12 ELISA. **a)** Fluorescence intensities of ThT assays showing aggregation of recombinant full-length tau over time. Seeds alone (tau aa256–368), buffer alone and full-length tau alone were used as controls. The signal for tau with seeds increases exponentially until app. 8 h of incubation (*n* = 3). Analysis of aggregates by **b)** Tau12-Tau12 ELISA assay and **c)** T22-Tau12 ELISA assay. Both methods yield a higher signal for tau with seeds after 48 h of incubation, which is abolished after boiling in SDS-containing buffer (*n* = 3). **d)** Dot blot analysis of native samples with T22 antibody: seeds alone, tau alone and tau with seeds at 0 h and 48 h. Two-way Anova for b and c: **, *p* < 0.01, ***, *p* < 0.001, ****, *p* < 0.0001

We first validated these methods using an in vitro aggregation assay with recombinant tau (2N4R). In parallel, we performed a Thioflavin T (ThT) binding assay to monitor the formation of beta sheet-containing structures as a readout for tau aggregation over time. Since full-length tau aggregation is a slow process in vitro, we added a pre-aggregated recombinant tau fragment encompassing the amino acids 256 to 368 as aggregation seeds [51]. As these seeds do not contain the Tau12 epitope, they should not interfere with the ELISA-based detection of full-length tau multimers. As expected, neither buffer nor seeds alone, nor full-length tau without seeds showed any increase in ThT signal over time (Fig. 3a). In contrast, the incubation of full-length tau with seeds led to an exponential increase in signal, slowing down after app. 8 h of incubation (Fig. 3a). Next, we performed an electrochemiluminescence ELISA with the Tau12-Tau12 pair to detect multimers. While we only observed a low baseline signal at the 0 h timepoint, the signal increased significantly for aggregated tau at 48 h (Fig. 3b). Interestingly, the signal of tau alone at 48 h also showed a significant increase, which was not detected by ThT assay. This suggests that compared to the ThT assay, the Tau12-Tau12 ELISA assay is more sensitive and detects additional non-monomeric tau species that may be either very small or do not contain β-sheet structures. Importantly, the signals from tau alone and tau with seeds at 48 h were completely abolished when the samples were boiled in SDS-containing buffer, confirming that the Tau12-Tau12 ELISA method can identify non-monomeric detergent-soluble tau species (Fig. 3b). In addition, the T22-Tau12 assay, which is expected to detect misfolded tau oligomers, showed a similar signal increase over time for both tau alone and tau with seeds, with the seeded aggregation reaction leading to the strongest signal, as expected (Fig. 3c). The boiling of samples with SDS-containing buffer abolished the signals, suggesting that the boiling process resolves oligomeric tau structures consistent with the Tau12-Tau12 assay. Moreover, dot blot analysis confirmed the time- and seeding-dependent generation of T22-positive oligomers (Fig. 3d).

**Fig. 4:**
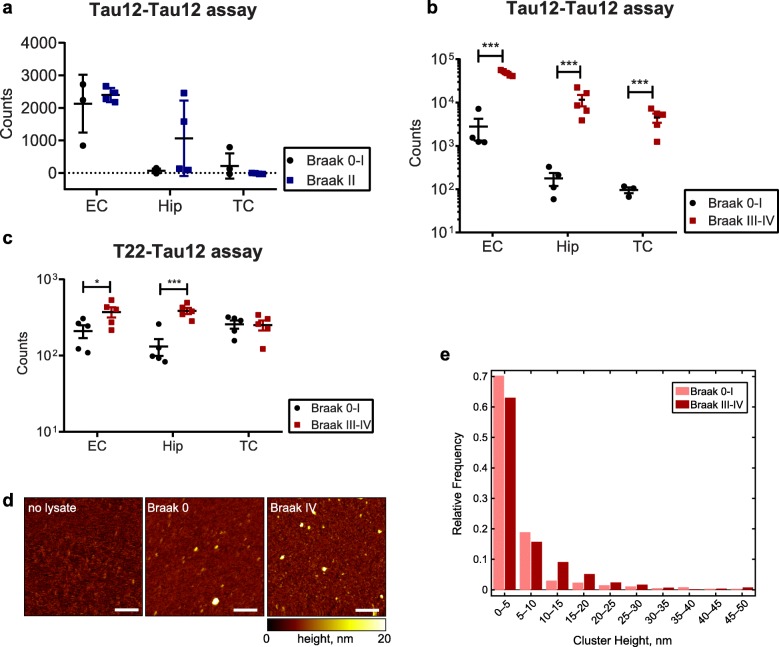
ELISA assays and atomic force microscopy (AFM) reveal more abundant tau oligomers in Braak III–IV ECs. Comparison of ELISA counts from Braak 0–I controls with **a)** Braak II entorhinal cortices (EC), hippocampi (Hip), and temporal cortices (TC) and **b, c)** Braak III–IV EC, Hip and TC, using Tau12-Tau12 (**a, b**) or T22-Tau12 (**c**) assays. **d)** Representative AFM images of eluates after Tau12 immunoprecipitation; left: eluate without brain lysate (negative control), middle: eluate of Braak 0 EC, right: eluate of Braak IV EC. Scale bars represent 200 nm. **e)** Relative frequencies of cluster heights detected from AFM scans of two Braak 0 and two Braak IV EC tissue samples shows increase of clusters > 10 nm in Braak IV samples. Number of clusters detected: Braak 0–I: 1343, Braak III–IV: 1053. Student’s t-tests: *, *p* < 0.05, ***, *p* < 0.001

Using the same Tau12-Tau12 setup, we then determined the presence of tau multimers in EC, Hip and TC tissues from donors classified as Braak stages 0–I, II, or III–IV (Fig. 4a and b). While we did not detect any significant differences between Braak 0–I and Braak II (Fig. 4a), all brain regions from Braak III–IV resulted in a significantly increased ELISA signal, suggesting that tau multimers are present (Fig. 4b). On the other hand, the analysis of the Braak III–IV brain regions with the T22-Tau12 assay showed that only EC and Hip contain significantly increased misfolded tau oligomers, suggesting that the T22-Tau12 assay may only detect a subset of the multimeric tau species recognized by the Tau12-Tau12 assay (Fig. 4c).

Tau oligomers associated with AD pathology have previously been reported to have diameters of 5–15 nm [50].To investigate the tau species in the EC of our Braak 0–I and Braak III–IV donors in more detail, we therefore immunoprecipitated tau with the Tau12 antibody and employed atomic force microscopy with quantitative image analysis. We found that for both Braak 0–I and III–IV, as well as for a negative control sample containing only Tau12 antibody without brain lysate, the atomically flat mica substrates are covered with an isotropic layer of molecules, leading to a topography with individual structures of up to 5 nm height. Clusters above 5 nm in height were only found in brain lysate samples. Here, the vast majority of clusters between 10 and 30 nm in height were detected in the Braak III–IV samples (Fig. 4d, e). This suggests that the significant increase of Tau12-Tau12 signal we observed in Braak III–IV EC may be due to these larger clusters.

**Fig. 5:**
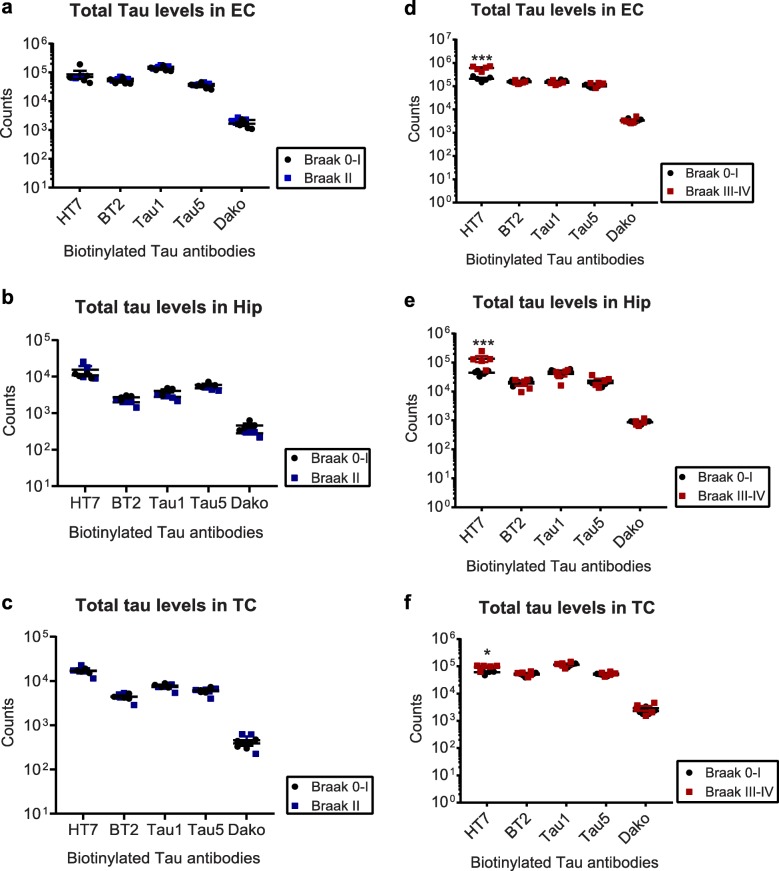
Total tau levels at different Braak stages in different brain regions. Total tau levels in **a, d)** Entorhinal cortices (EC), **b, e)** Hippocampi (Hip), and **c, f)** Temporal Cortices (TC) from Braak II (**a–c**) and Braak III–IV (**d–f**) samples, along with their age-matched Braak 0–I controls. Student’s t-tests: *, *p* < 0.05, ***, *p* < 0.001

We then asked whether the different amounts of multimeric structures detected by Tau12-Tau12 or T22-Tau12 assays were due to different total levels of tau in the detergent-soluble fraction, and used six different total tau antibodies (HT7, BT2, Tau1, Tau5 and Dako-Tau) raised against different domains of tau as capture antibodies and Tau12 as detection antibody (Fig. 5). While total tau levels in all Braak 0–I and Braak II samples did not show any differences (Fig. 5a-c), all three brain regions from Braak III–IV donors exhibited an increased signal only with HT7 as capture antibody but not with BT2, Tau1, Tau5 and Dako-Tau antibodies (Fig. 5d-f).

**Fig. 6:**
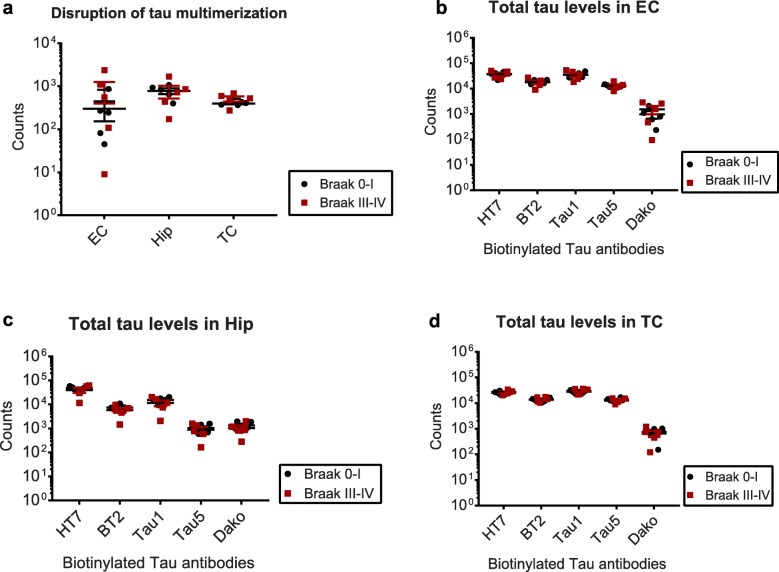
Tau multimers can be disrupted by boiling in SDS-containing buffer. **a)** Comparison of tau multimer levels in entorhinal cortices, hippocampi, and temporal cortices between Braak 0–I and Braak III–IV after boiling with SDS-containing buffer. Comparison of total tau levels in **b)** Entorhinal cortices (EC) **c)** Hippocampi (Hip) and **d)** Temporal cortices (TC) between Braak 0–I and Braak III–IV

Since these changes may be caused by tau multimers in the Braak III–IV samples, we next decided to assess whether boiling in SDS-containing buffer would resolve the difference to Braak 0–I tissue, similar to what we found for aggregates generated from recombinant tau protein (Fig. 3b and c). Indeed, the denaturation treatment abolished the difference in Tau12-Tau12 ELISA signal between Braak 0–I and Braak III–IV samples for all three brain regions (Fig. 6a). Similarly, also the previously seen difference in HT7-Tau12 signal (Fig. 5d-f) was not observed when boiled Braak 0–I and Braak III–IV EC, Hip and TC tissue samples were compared (Fig. 5b-d). Signals for all other total tau antibody combinations stayed similar between Braak stages, suggesting that the differences in Tau12-Tau12 and HT7-Tau12 signal in native samples were a result of tau multimerization, while the other antibody pairs were not as sensitive to aggregation state. Furthermore, these findings suggest that overall tau levels were not different between Braak stages in the Triton-soluble extracts.

### Five consistently increased tau PTMs differentiate Braak stages 0–I and III–IV

**Fig. 7:**
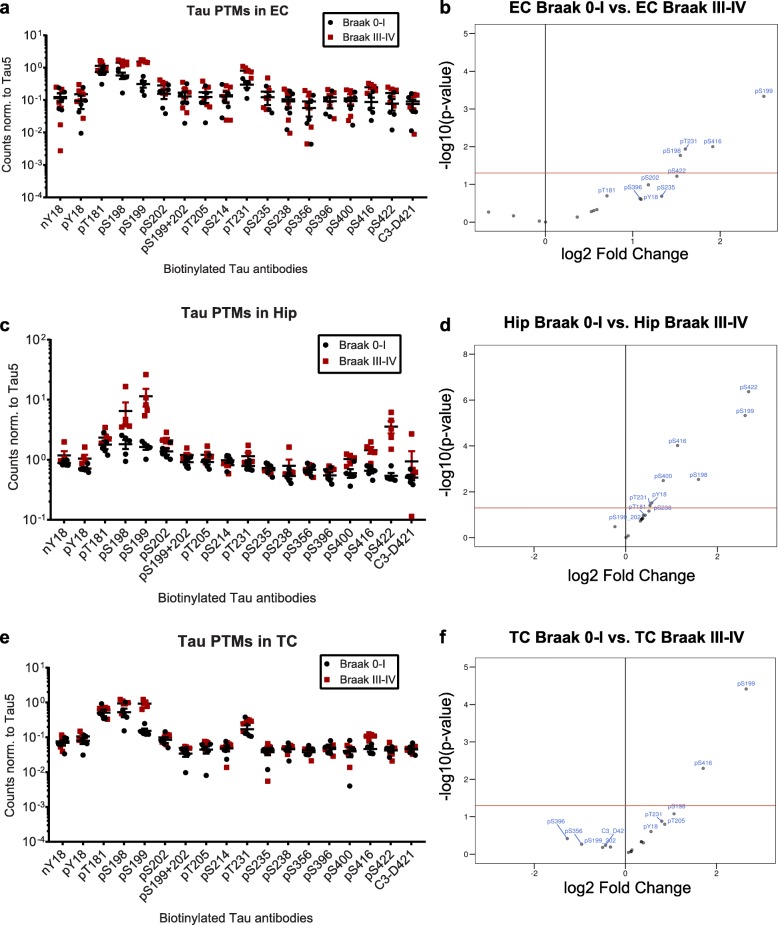
Tau PTMs in denatured Braak III–IV samples. Normalized PTM signals from **a)** Entorhinal cortices (EC), **c)** hippocampi (Hip), and **e)** temporal cortices (TC) of Braak stages 0–I and III–IV. **b, d, f)** Corresponding fold changes (log2) versus significance (−log10(*p*-value)) of the changes. Phosphorylation at the sites above the red line, which corresponds to *p*-value = 0.05, is significantly higher in Braak III–IV samples

Since we had detected high levels of tau oligomers in all Braak III–IV samples, we next boiled the lysates with SDS-containing buffer and re-analyzed the PTM levels. Among the PTMs with previously observed increases (Fig. 2 and Additional file 3: Figure S2), this treatment dramatically reduced the differences between Braak stages (Fig. 7): In denatured samples, we found that the sites pS198, pS199, pT231, pS416 were significantly higher in the EC of Braak III–IV compared to Braak 0–I samples (Fig. 7a, b), in Hip tissue pY18, pS198, pS199, pT231, pS400, pS416 and pS422 were significantly increased at Braak stages III–IV (Fig. 7c, d), and in TC sites pS199 and pS416 were elevated in Braak III–IV compared to Braak 0–I (Fig. 7e, f).

Since there was a lot of overlap with regards to which PTMs were dysregulated in the different tissues, we next generated a linear model that takes changes in tau PTMs in four sample types into account: EC from Braak stage II, as well as EC, Hip and TC from Braak stages III–IV, in comparison to their respective Braak 0–I controls. This comparison revealed the sites pS198, pS199, pT231, pS416 and pS422 to be significantly (adj. *p*-value < 0.01) increased over control in our cohort (Table 3).

**Table 3:** Tau PTM events increased in at least one Braak III–IV tissue. Statistical significance was determined with Omnibus test

PTM events	*P*-value	adjusted *P*-value
pS198	0.0007346	0.003306
pS199	0.0000003316	0.000005968
pT231	0.001081	0.003891
pS416	0.0004662	0.002797
pS422	0.00004812	0.0004331

### iPSC-derived neurons derived from sporadic and familial AD patients do not exhibit tau multimerization or aberrant tau PTMs

iPSC-derived neurons are an increasingly popular system to model neurodegenerative diseases in vitro, and lines generated from patient cells should in theory allow for disease modeling even in the absence of a familial mutation [52]. Nevertheless, these neuronal cultures represent an early developmental stage and there are conflicting reports as to whether AD-related tau phenotypes can be observed [52–54]. We therefore decided to investigate whether Braak-stage dependent changes in tau PTMs observed in brain tissue can be recapitulated in iPSC-derived neurons.

**Fig. 8:**
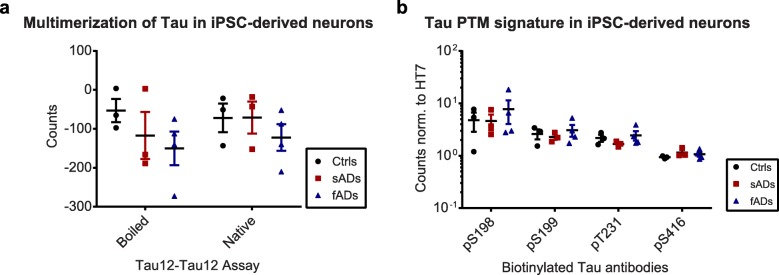
iPSC-derived neurons do not recapitulate the tau PTM signature. **a)** Analysis of multimers by Tau12-Tau12 electrochemiluminescence assay with and without boiling of lysates from controls, familial AD (fAD) and sporadic AD (sAD) neurons with SDS. **b)** Normalized PTM signals (pS198, pS199, pT231 and pS416). None of the observed changes were significant (*p* > 0.05, t-tests)

To this end, we generated cortical neurons from three control iPSC lines, three sporadic AD (sAD) and four familial AD (fAD) iPSC lines, each from a different donor fibroblast culture (Additional file 1: Table S1 and Additional file 4: Figure S3, [47]). From each line, we performed at least two independent differentiation rounds to assess variability. As our first readout, we checked whether tau multimers were present in sAD or fAD cells. Using the Tau12-Tau12 ELISA assay, we did not observe a consistent signal for any of the lines, and no change in signal was observed when lysates were boiled in SDS-containing buffer (Fig. 8a). This is in agreement with previous reports showing that the iPSC-derived neurons do not contain any forms of multimeric or aggregated tau in the absence of additional triggers such as tau mutations, overexpression or seeding [55, 56]. Similarly, no significant differences were observed between control, sAD and fAD lines when comparing the levels of pS198, pS199, pT231 and pS416 – four sites that were significantly increased in brain tissues from Braak III–IV donors (Fig. 8b). Taken together, these findings suggest that the generation of iPSC-derived neurons with a cortical identity is not sufficient to consistently recapitulate changes in tau multimerization and PTM status that is observed in post-mortem patient tissues.

### Three PTMs correlate with tau multimerization

Tau hyperphosphorylation increases its aggregation propensity in vitro [57, 58], and PHF-tau isolated from AD patient brains is heavily phosphorylated [59]. However, it remains unclear whether aggregation in vivo is driven by an increase in specific PTMs on soluble tau. We therefore tested whether the changes in tau PTMs observed in Braak III–IV brain tissues correlate with tau multimerization and the formation of misfolded oligomers. To this end, we performed a Spearman correlation analysis between the state of tau obtained by Tau12-Tau12 and T22-Tau12 assay, and fold changes of all PTM sites for each individual denatured sample (Table 4). Multiple sites showed a strong (r > 0.5) and significant (*p* < 0.05) correlation. The Tau12-Tau12 multimerization assay revealed that in the EC, phosphorylation events at sites S198, S199, T231 and S416 correlated with multimerization. For Hip, pY18, pS198, pS199, pS202, pT205, pS238, pS396, pS400, pS416 and pS422 showed a positive correlation with tau multimerization, while a negative correlation was observed for pS214. Lastly, for TC, the sites pT181, pS198, pS199, pT231, pS416 correlated with tau multimerization. The T22-Tau12 oligomerization assay on the other hand did not reveal any correlation in EC, but in Hip the sites nY18, pY18, pS198, pS199, pT205, pS396, pS400, pS416 and pS422 showed a positive correlation (Table 4). Since no changes were detected with the T22-Tau12 ELISA in Braak III–IV TC (Fig. 4c), this tissue was not included in the correlation analysis for misfolded oligomers.

**Table 4:**
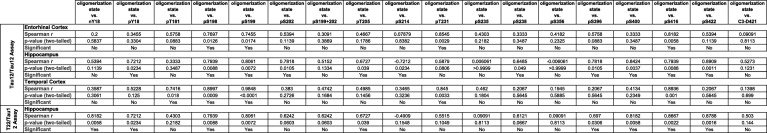
Correlation analysis of tau PTMs and oligomerization state

**Fig. 9:**
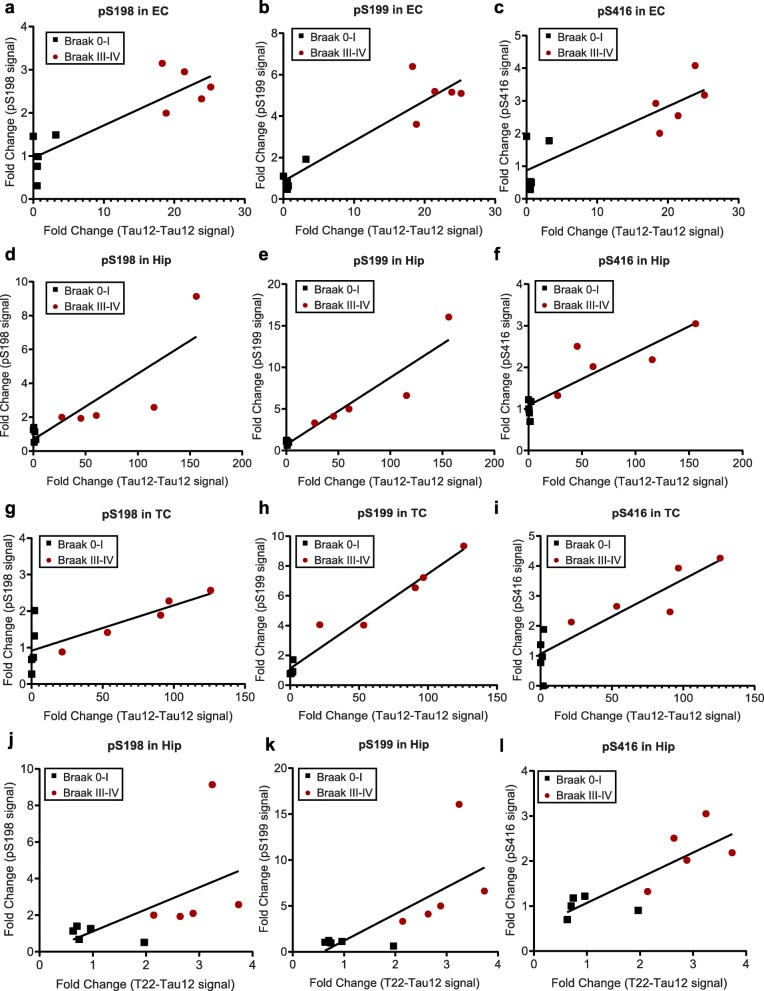
Correlation of tau oligomerization with pS198, pS199, and pS416 fold changes in all brain regions. Spearman correlation of the fold changes in Tau12-Tau12 signal with the fold changes (black squares: Braak 0–I / average (Braak 0–I); red circles: Braak III–IV / average (Braak 0–I)) of **a)** pS198, **b)** pS199 and **c)** pS416 in entorhinal cortex (EC), **d)** pS198, **e)** pS199 and **f)** pS416 in hippocampus (Hip), **g)** pS198, **h)** pS199 and **i)** pS416 in temporal cortex (TC) and Spearman correlation of the fold changes in T22-Tau12 signal with the fold changes (black squares: Braak 0–I / average (Braak 0–I), red circles: Braak III–IV / average (Braak 0–I)) of **j)** pS198, **k)** pS199 and **l)** pS416 in hippocampus (Hip). Results of the statistical analysis are summarized in Table 4

Among these phosphorylation events, pS198, pS199 and pS416 were consistently correlated with an increase in Tau12-Tau12 or T22-Tau12 ELISA signal in all brain regions analyzed (Table 4 and Fig. 9). Phosphorylation at these three sites also emerged as significantly increased in our analysis of PTM level differences (Table 3). Increases in pT231 levels, on the other hand, only correlated with multimerization in EC and TC, while the increase in pS422 correlated with the Tau12-Tau12 and the T22-Tau12 signals only in Hip (Table 4). These findings suggest that three specific PTM sites are not only increased at early Braak stages, but their presence also strongly correlates with the formation of soluble tau multimers and misfolded oligomers, a marker of tau toxicity in AD.

## Discussion

While tau dysfunction and toxicity has been linked to the formation of soluble oligomeric structures, these early intermediates are difficult to study in complex samples such as human brain. Therefore, much is known about PTMs and in particular tau phosphorylation on NFTs, but it is unclear whether the same sites are already differentially modified on soluble tau before aggregation. In this study we present a systematic analysis of PTM changes on soluble tau during early AD from human brain samples. While total tau levels are comparable between disease stages in these fractions, we do observe a strong shift particularly in tau phosphorylation during the progression from Braak stages 0–I to III–IV. Since many phospho-sites demonstrate an increased signal in native, but not in denatured Braak III–IV samples, our data suggest that phospho-tau molecules form multimers together with non-modified tau, which thus provides additional binding sites for the Tau12 detection antibody. Interestingly, the sites showing a consistent increase in denatured samples are different from those that are traditionally used to stain NFTs and perform immunohistochemical Braak staging such as AT8 (pS202/pT205). However, despite the presence of antibodies against these phospho-sites in our panel, we did not observe an increase for their epitopes in the Triton-soluble fraction of Braak III–IV brains, although their signals did correlate with tau oligomerization in Hip tissue. This is in line with previous findings that the phospho-tau pattern differs during the development of NFTs, with specific phospho-sites being associated with pre-neurofibrillary tangles, intra- or extra-neuronal neurofibrillary tangles [60]. AT8 staining in particular is strongly associated with fibrillar aggregates [22], but has been observed in individuals as young as 20 years of age [61]. Braak and colleagues have therefore proposed that the occurrence of clinical AD symptoms may require synergistic effects between this age-dependent tauopathy and an additional insult [61]. Our results show a clear shift towards an increase of both tau multimerization and specific tau PTMs at Braak stages III–IV in the EC. Since AT8 staining in the EC is a defining feature already at Braak II, this suggests that tau pathology still increases in this brain region with disease progression.

Although most individuals at Braak III–IV are still clinically asymptomatic, we find biochemical manifestations of AD such as increased tau multimerization and phosphorylation even in the TC, which at this stage is largely AT8 negative. Importantly, we define a signature of three tau PTMs that is consistently increased and associated with multimerization throughout the EC, Hip and TC. Among the PTM events we identified, only pT231 has been previously linked to pre-tangle structures and was found increased at Braak stages corresponding to early disease (III–IV) [60, 62]. However, these studies were performed with a smaller antibody panel and by immunostaining, which is inherently less quantitative than ELISA. Furthermore, both pS199 and pT231 are increased in the cerebrospinal fluid (CSF) of AD patients and are strongly increased in our samples, while pT181, a third commonly used CSF biomarker [63], did not differ between Braak stages in our study. pS416 and pS422, on the other hand, are likely too far at the tau C-terminus to be present on the truncated forms of tau detectable in CSF [64].

pS416 and pS422 were both previously described as being phosphorylated on synaptic tau in both human patients and mouse models [65–67]. pS422 in particular has been targeted by a passive immunization strategy in triple transgenic mice (TauPS2APP mice, [65]), and data from the same mouse model suggest that this phosphorylation event is promoted by the presence of amyloid plaques [66]. The fact that tau pS422 is most prominently changed in the Hip in our analysis therefore makes it tempting to speculate that this form of tau may actually be located synaptically in projections from excitatory pyramidal neurons in the EC, which are the most vulnerable neuron population at early stages of AD [68, 69].

Misfolded tau oligomers are thought to be a major source of neuronal dysfunction in AD, and we detected increased T22 signal in EC and Hip tissues, which also show the most alterations in PTMs at Braak stage III–IV. The increase in phosphorylation at the sites of our PTM signature may therefore alter the oligomerization and/or aggregation propensity of tau molecules, although such a connection still has to be formally demonstrated. Our correlation analysis between tau multimerization and PTM fold changes showed that pS198, pS199 and pS416 correlate with tau multimerization in all brain regions. A correlation with pT231 levels was only observed in EC and TC, while pS422 correlates with Tau12-Tau12 and T22 signals in Hip, where it is also most prominently increased. This argues against non-specific, general hyperphosphorylation of tau as a trigger of pathology and may thus be different from the physiological phosphorylation events occurring during development, anesthesia and hypothermia [20]. However, the factors responsible for the specific changes we observed remain unknown. Potential candidate enzymes include the kinases GSK3B, TTBK1, CAMK, PKA, CDK5 and the phosphatases PP2A and PP5 (www.tauptm.org) [19].

While further studies in human brain tissues are hampered by factors that influence enzymatic activities such as postmortem interval times [70], such studies are much easier to perform in model systems, and the use of iPSC-derived neurons for neurodegenerative disease research has revolutionized the field in the last years [71]. However, when we studied the tau PTM signature in iPSC-derived neurons from sporadic and familial AD patients, we found that the pattern we observed in human brains was not recapitulated, which might be due to their developmental immaturity and the absence of tau oligomerization in these cells. Developing cellular models for AD and especially to study tau is challenging [56]. Despite many advantages, iPSC-derived neurons have the caveat that they express only one out of six isoforms of tau [53], and reprogramming results in the loss of aging factors, which may affect disease pathology [54, 72]. Using isogenic controls can be helpful to discern subtle disease phenotypes, however this is not an option for sporadic diseases without a single genetic cause [52].

For tau phosphorylation, previous studies have yielded variable results with some, but not all sporadic AD lines showing an increase [73, 74]. For familial AD, tau phenotypes have been reported for lines containing APP, but not presenilin mutations [75, 76]. As three out of our four familial AD lines had PS1 mutations, this may be a reason for the lack of tau phenotypes in our cultures. Furthermore, a new study has also revealed that inter-laboratory variability is the largest source of failed reproducibility of experiments performed by iPSC-derived neurons [77].

With the advent of more complex culture systems such as 3D and co-culture models, it remains to be seen if iPSC technology can yield more robust phenotypes for sporadic and age-dependent disease in the future.

## Supplementary information

**Additional file 1: Table S1.** List of iPSC-derived neurons used in the study.

**Additional file 2: Figure S1.** Non-phospho tau PTMs do not change in Braak II. Normalized tau PTM signals (nY18, nY29, Ack280, meK311, C3-D421) in Braak II **a)** Entorhinal cortices (EC) **b)** Hippocampi (Hip) and **c)** Temporal cortices (TC) compared to Braak 0–I controls. None of the observed changes were significant (*p* > 0.05, t-tests).

**Additional file 3: Figure S2.** Specific increase in tau proteolysis at D421 and nitration at Y18 in native Braak III–IV compared to Braak 0–I samples. a, b, c) Normalized tau PTM signals (nY18, nY29, Ack280, meK311, C3-D421) from native Braak III–IV and Braak 0–I entorhinal cortices, hippocampi and temporal cortices. Student’s t-tests: *, *p* < 0.05, **, *p* < 0.01, ***, *p* < 0.001 (t-tests).

**Additional file 4: Figure S3.** Differentiation of iPSCs from control, fAD and sAD donors to cortical neurons. Representative microscopy images of iPSC-derived neurons stained for neuronal markers **a)** MAP 2 (red), GABA (green) **b)** vGlut (red), NeuN (green) **c)** MAP 2 (green), Tau12 (red) and **d)** Tuj1 (green) and Tbr1 (red) and DAPI for nuclei (blue). Scale bars represent 50 μm for all images.

## Data Availability

The datasets during and/or analysed during the current study available from the corresponding author on reasonable request.
