# Supplementary figures and images for: A post-translational modification signature defines changes in soluble tau correlating with oligomerization in early stage Alzheimer’s disease brain
Source: Acta Neuropathol Commun. 2019 Dec 3;7:192. doi: 10.1186/s40478-019-0823-2 (PMC6892178; doi:10.1186/s40478-019-0823-2)

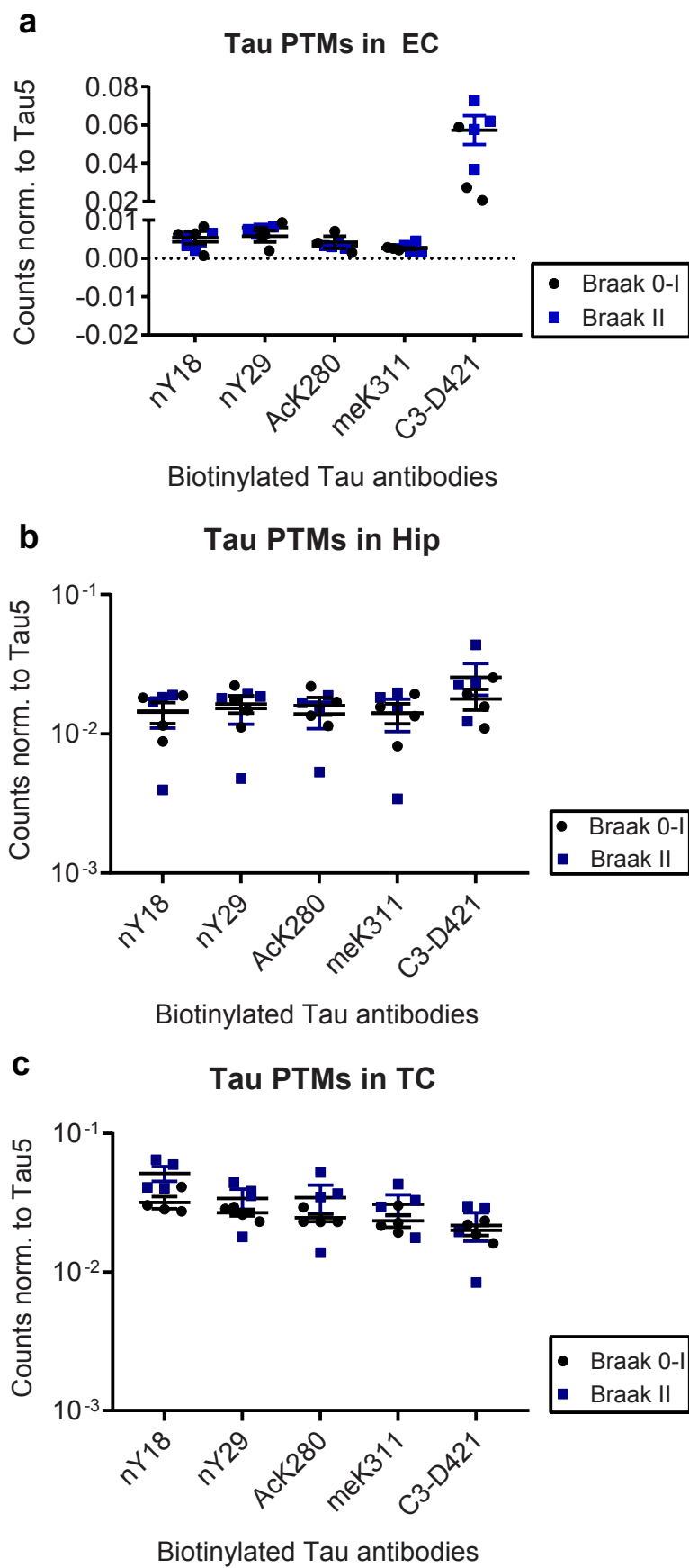

Supplementary Figure 1

Supplement: Supplementary file 2 — Additional file 2: Figure S1. Non-phospho tau PTMs do not change in Braak II. Normalized tau PTM signals (nY18, nY29, Ack280, meK311, C3-D421) in Braak II a) Entorhinal cortices (EC) b) Hippocampi (Hip) and c) Temporal cortices (TC) compared to Braak 0–I controls. None of the observed changes were significant (p > 0.05, t-tests). [file 40478_2019_823_MOESM2_ESM.pdf]

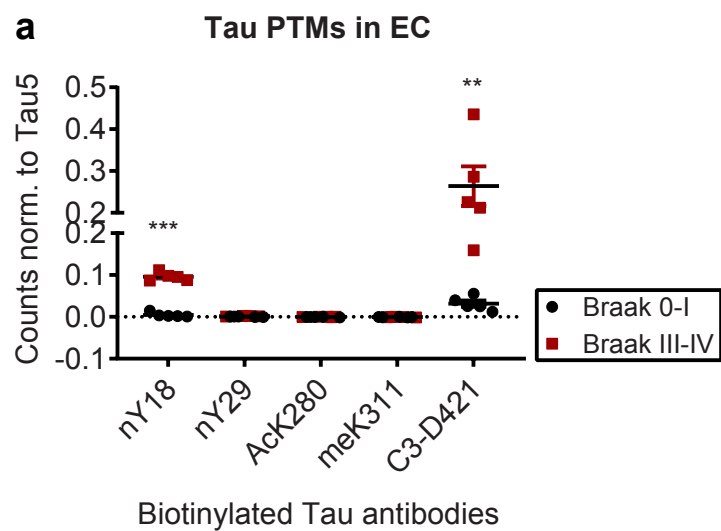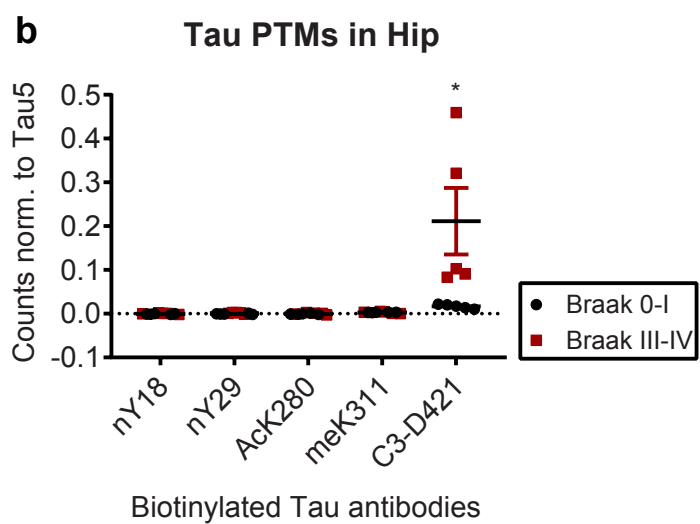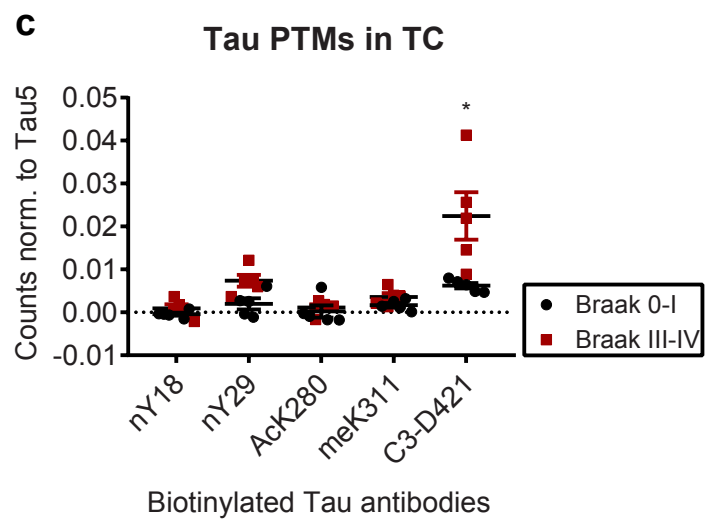

Supplementary Figure 2

Supplement: Supplementary file 3 — Additional file 3: Figure S2. Specific increase in tau proteolysis at D421 and nitration at Y18 in native Braak III–IV compared to Braak 0–I samples. a, b, c) Normalized tau PTM signals (nY18, nY29, Ack280, meK311, C3-D421) from native Braak III–IV and Braak 0–I entorhinal cortices, hippocampi and temporal cortices. Student’s t-tests: *, p < 0.05, **, p < 0.01, ***, p < 0.001 (t-tests). [file 40478_2019_823_MOESM3_ESM.pdf]

**a** MAP2/GABA/DAPI

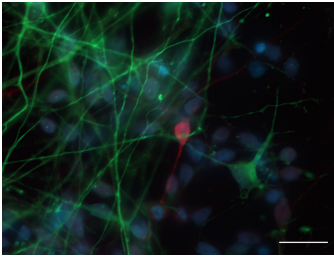

**b** NeuN/vGLUT/DAPI

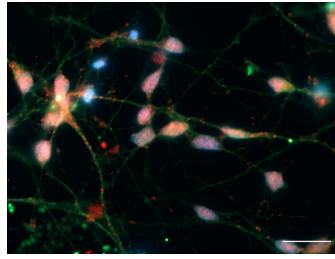

**c** MAP2/Tau12/DAPI

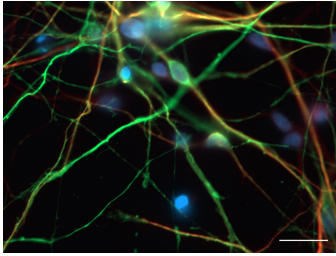

**d** Tuj1/Tbr1/DAPI

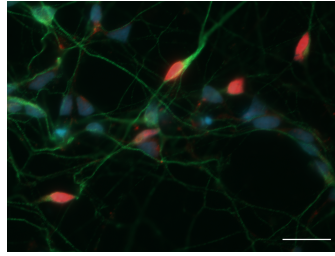

Supplementary Figure 3

Supplement: Supplementary file 4 — Additional file 4: Figure S3. Differentiation of iPSCs from control, fAD and sAD donors to cortical neurons. Representative microscopy images of iPSC-derived neurons stained for neuronal markers a) MAP 2 (red), GABA (green) b) vGlut (red), NeuN (green) c) MAP 2 (green), Tau12 (red) and d) Tuj1 (green) and Tbr1 (red) and DAPI for nuclei (blue). Scale bars represent 50 μm for all images. [file 40478_2019_823_MOESM4_ESM.pdf]
